# Oral administration of EPA-rich oil impairs collagen reorganization due to elevated production of IL-10 during skin wound healing in mice

**DOI:** 10.1038/s41598-019-45508-1

**Published:** 2019-06-24

**Authors:** Beatriz Burger, Carolina M. C. Kühl, Thamiris Candreva, Renato da S. Cardoso, Jéssica R. Silva, Bianca G. Castelucci, Sílvio R. Consonni, Helena L. Fisk, Philip C. Calder, Marco Aurélio R. Vinolo, Hosana G. Rodrigues

**Affiliations:** 1 Laboratory of Nutrients and Tissue Repair, School of Applied Sciences, University of Campinas, Limeira, SP Brazil; 2 Department of Genetics, Evolution, Microbiology and Immunology, Institute of Biology, University of Campinas, Campinas, SP Brazil; 3 Department of Biochemistry and Tissue Biology, Institute of Biology, University of Campinas, Campinas, SP Brazil; 4 Human Development & Health, Faculty of Medicine, University of Southampton, Southampton, United Kingdom; 5 NIHR Southampton Biomedical Research Centre, University Hospital Southampton NHS Foundation Trust and University of Southampton, Southampton, United Kingdom

**Keywords:** Immunology, Acute inflammation

## Abstract

Wound healing is an essential process for organism survival. Some fatty acids have been described as modulators of wound healing. However, the role of omega-3 fatty acids is unclear. In the present work, we investigate the effects of oral administration of eicosapentaenoic acid (EPA)-rich oil on wound healing in mice. After 4 weeks of EPA-rich oil supplementation (2 g/kg of body weight), mice had increased serum concentrations of EPA (20:5ω-3) (6-fold) and docosahexaenoic acid (DHA; 22:6ω-3) (33%) in relation to control mice. Omega-3 fatty acids were also incorporated into skin in the EPA fed mice. The wound healing process was delayed at the 3^rd^ and 7^th^ days after wounding in mice that received EPA-rich oil when compared to control mice but there was no effect on the total time required for wound closure. Collagen reorganization, that impacts the quality of the wound tissue, was impaired after EPA-rich oil supplementation. These effects were associated with an increase of M2 macrophages (twice in relation to control animals) and interleukin-10 (IL-10) concentrations in tissue in the initial stages of wound healing. In the absence of IL-10 (IL-10^−/−^ mice), wound closure and organization of collagen were normalized even when EPA was fed, supporting that the deleterious effects of EPA-rich oil supplementation were due to the excessive production of IL-10. In conclusion, oral administration of EPA-rich oil impairs the quality of wound healing without affecting the wound closure time likely due to an elevation of the anti-inflammatory cytokine IL-10.

## Introduction

Skin is the first line of the body’s immunological defense against physical, chemical or biological aggression from the external environment^1,2^. Mammalian skin is divided into epidermis that contains keratinocytes, and dermis composed of fibroblasts and extracellular matrix (ECM). In ECM, collagen, laminins, and elastic fibers provide flexibility; whereas glycosaminoglycan, proteoglycans and hyaluroan stabilize growth factors and the three-dimensional space by their high water-binding ability^3^. Skin has appendages (hair follicles, sebaceous and sweat glands) and nerves, sensory corpuscles and vasculature. The dermis and skin appendages have important roles in the reepithelialization process because they provide nutritional and mechanical support and supply progenitor cells for the restoration of the epidermis after wounding^4^. Skin homeostasis is provided by the epidermis, dermis and ECM through the interaction between keratinocytes, fibroblasts and resident immune cells, such as neutrophils, macrophages and T lymphocytes^5^.

After external skin damage, the organism needs to repair itself quickly to avoid dehydration, blood loss and the entrance of harmful microorganisms. Thus a wound healing process initiates and involves an intrinsic and coordinated cascade of events divided into 3 phases: inflammation, formation of granulation tissue and maturation^6–8^. The process is orchestrated by different cell types such as platelets, neutrophils, macrophages, endothelial cells and fibroblasts, as well as protein (cytokines, growth factors) and lipid (prostaglandins, leukotrienes, thromboxanes and lipoxins) mediators^9^.

Once a wound is closed, the immature scar can move onto the final remodeling phase. The ECM is important in the maintenance of the structure, function, and signaling of tissues. Then, the ECM molecules reorganize to a cross-linked mode in an attempt to restore skin functionality. Targeting components of the ECM during wound repair provides an attractive approach to avoid hypertrophic and keloid scars^3^.

Despite the biological processes that act to promote wound healing, a significant proportion of the global population suffers from hard to heal wounds, including people with diabetes and elderly individuals. Wounds may require multifactorial treatment and combinations of antimicrobials, protective barriers and skin grafts may be needed to achieve successful healing. Topical treatment with fatty acids appears to be useful for maintenance of hydration and elasticity of the skin, preventing the entrance of microorganisms and water loss to the external environment^10^.

Our group has demonstrated that oral administration of linoleic acid (LA, 18:2ω-6) at a dose of 0.22 g/kg bw for 10 days promoted wound healing in rats, because it improved the inflammatory response and angiogenesis^8,11,12^. The effects of omega-3 (ω-3) fatty acids on wound healing are less clear^6^.

Eicosapentaenoic acid (EPA, 20:5 ω-3) and docosahexaenoic acid (DHA; 22:6 ω-3) are the major ω-3 polyunsaturated fatty acids (PUFAs) found in fish oils^13,14^. These fatty acids are found in skin in low concentrations, especially in individuals consuming a Western diet characterized by high amounts of ω-6 over ω-3 PUFA sources^14,15^. Increased ω-3 PUFA intake leads to the incorporation of these fatty acids into phospholipids of cell membranes^16^ and, the consequent accumulation of metabolites derived from them in the epidermis which explains the beneficial effects of ω-3 fatty acids in cutaneous inflammation, such as psoriasis^17^.

Although EPA and DHA are classified in the same family, they have different effects on leukocyte functions, insulin-secreting cells and endothelial cells because they act differently on the physicochemical properties of membranes, on the intracellular signaling pathways and in gene expression control^18,19^. To our knowledge, few studies have investigated the effects of oral administration of EPA-rich oil on wound healing in mice. The hypothesis of this study was that oral supplementation with EPA-rich oil will delay wound healing due to its anti-inflammatory effects.

## Results

### EPA-rich oil supplementation increases ω-3 fatty acid concentrations in serum and skin of mice

Animals were orally supplemented daily with EPA-rich oil (2 g/kg of bw) during 4 weeks (Supplemental Fig. 1A).

The assessment of nutritional parameters such as food and water intake; and body weight (bw) showed that EPA-rich oil supplementation did not induce changes in the general health of the mice (Supplemental Fig. 1B). Furthermore, these results indicate that any effect on the wound healing process would not be due to nutritional changes.

**Figure 1:**
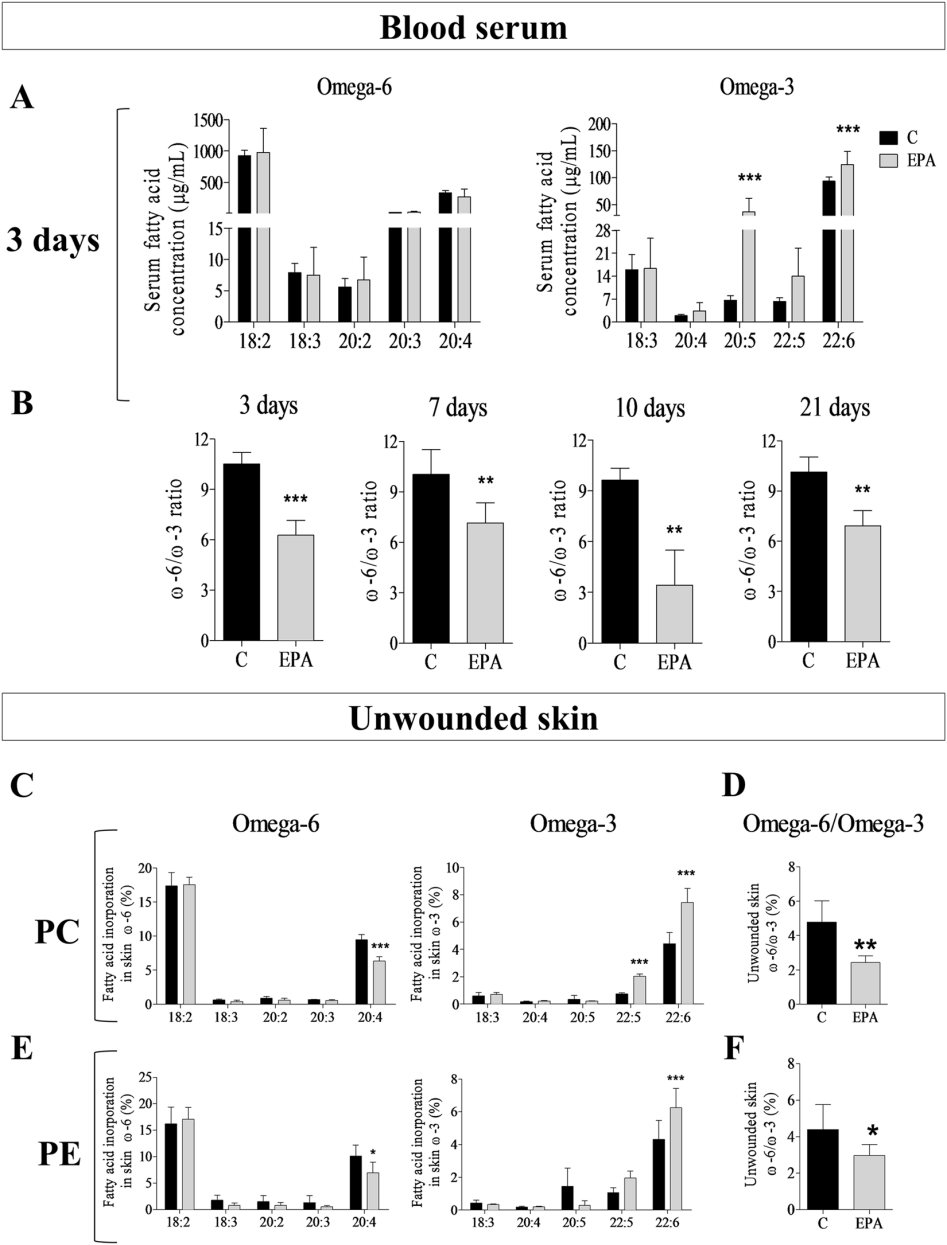
Fatty acid composition of serum and unwounded skin throughout experiments in the control group (C, black bar) and EPA-group (EPA, grey bar). (**A**) Omega-3 and omega-6 concentrations in serum. (**B**) Omega-6/omega-3 ratio in serum. (**C**) Omega-3 and omega-6 content of skin phosphatidylcholine (PC). (**D**) Omega-6/omega-3 ratio in skin phosphatidylcholine (PC). (**E**) Omega-3 and omega-6 content of skin phosphatidylethanolamine (PE). (**F**) Omega-6/omega-3 ratio in skin phosphatidylethanolamine (PE). (n = 5–13 animals/group). Healthy mice were supplemented daily with EPA-rich oil (2 g of EPA-rich oil/kg bw) for 4 weeks and the serum and skin were sampled immediately prior to induction of the skin lesion and during the wound healing process. The percentage contribution of each fatty acid to the total fatty acid pool in each fraction was determined by gas chromatography. Values are expressed as mean ± SD. *p < 0.05; **p < 0.01, ***p < 0.001 indicates significant differences in relation to the control as indicated by Two-Way analysis of variance (ANOVA) and Bonferroni post-test (**A**,**C**,**E**) or test *t* and Mann Whitney post-test (**B**,**D**,**F**). The fractions analyzed were: 18:2 (ω-6) – Linoleic acid (LA); 18:3 (ω-6) – Gamma-linolenic acid (GLA); 20:2 (ω-6) – Eicosadienoic acid; 20:3 (ω-6) – Dihomo-gamma-linolenic acid (DGLA); 20:4 (ω-6) – Arachidonic acid (AA); 18:3 (ω-3) – Alpha linolenic acid (ALA); 20:4 (ω-3) – Eicosatetrenoic acid (ETA); 20:5 (ω-3) – Eicosapentaenoic acid (EPA); 22:5 (ω-3) – Docosapentaenoi acid (DPA); 22:3 (ω-3) – Docosahexaenoic acid (DHA).

After 4 weeks of feeding the oil, a wound was induced in the animal’s dorsum and blood samples were collected at specific time points (3, 7, 10 and 21 days) for the analysis of serum fatty acids (Fig. 1A,B). During the inflammatory phase (3 days), we observed a 6-fold increase in EPA (20:5ω-3) and a 33% increase in DHA (22:6ω-3) concentrations in the EPA group when compared to the control animals (C) (Fig. 1A). No alterations were observed in the concentrations of ω-6 fatty acids in serum. We also calculated the ω-6/ω-3 ratio and the EPA group had a lower ratio throughout the wound healing process (Fig. 1B).

Considering that alterations in skin fatty acid (FA) composition by diet is secondary to the effects of diet on FA composition in circulating blood^15^ our next step was to evaluate the fatty acid composition of skin after oral administration of EPA-rich oil. As shown in Fig. 1C–F, the EPA group had higher incorporation of ω-3 fatty acids, mainly docosapentaenoic acid (DPA, 22:5ω-3) and DHA into the phosphatidylcholine (PC) fraction of skin and higher incorporation of DHA into the phosphatidyletanolamine (PE) fraction in relation to the Control group. There was lower incorporation of arachidonic acid (AA; 20:4ω-6) in both fractions in the EPA group when compared to the Control group. The ω-6/ω-3 ratio was also lower in both skin lipid fractions in the EPA group. Thus, the experimental protocol used was effective in modifying both the serum and skin fatty acid composition throughout the experiments.

### EPA-rich oil supplementation impaired the wound healing process

To assess the effects of oral administration of EPA-rich oil on wound closure, mice were subjected to surgical full-thickness removal of 1 cm^2^ of skin, in the dorsal region, and then monitored during 21 days (Supplemental Fig. 1A).

**Figure 2:**
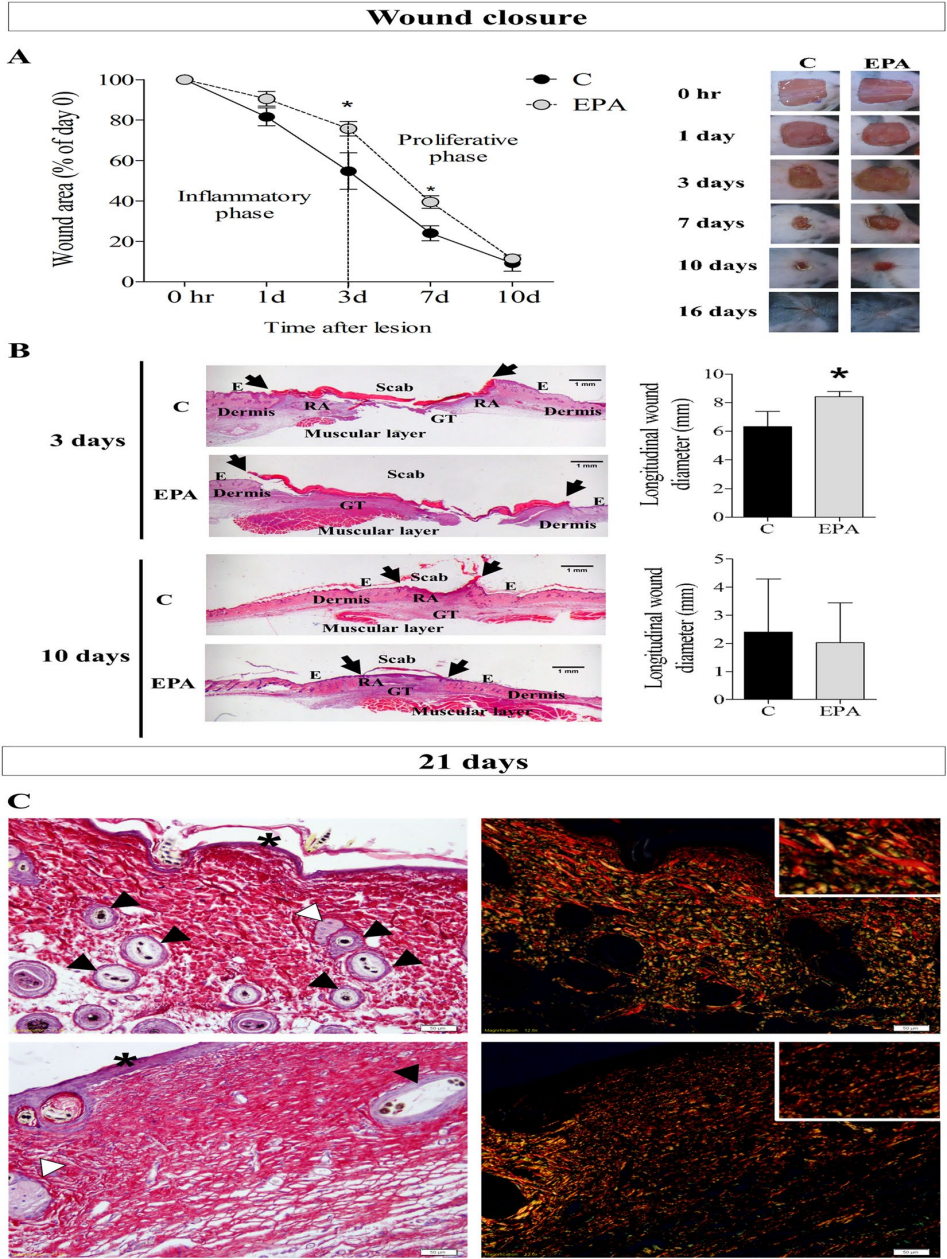
Wound closure and dermal architecture of late granulation tissue (21 days after lesion) in the control group (C, Black bar) and EPA-group (EPA, grey bar). (**A**) Wound area percentages during the experimental period and representative photos of wounds during the experiment (n = 7–9 animals/group). Values are expressed as mean ± SEM. *p < 0.05 indicates significant differences in relation to the control as indicated by two-way analysis of variance (ANOVA) and Bonferroni post-test. (**B**) Histological sections were stained with hematoxylin and eosin. Progression of the re-epithelium is indicated by arrows and graphs of wound diameter (mm) on skin harvested at 3 and 10 days (n = 4–5 animals/group). Scale bar: 1 mm. Values are expressed as mean ± SD. *p < 0.05 indicates significant differences in relation to the control as indicated by test *t* and Mann Whitney post-test. (**C**) Representative photomicrographs of skin stained with picrosirius and hematoxylin. The examination without (left) and with (right) polarized light revealed the organization and heterogeneity of collagen fiber orientation, squamous stratified epithelium (asterisk) and bulbs of the hair follicles (black arrowhead) and sebaceous glands (white arrowhead) in 21^st^ day after lesion induction (n = 3–5 animals/group). Scale bar = 50 µm.

The supplementation with EPA-rich oil delayed tissue repair on the 3^rd^ and 7^th^ days after wounding in relation to the control group, based on wound area percentage (Fig. 2A). The histological analyses of wounds revealed that the EPA group presented a larger longitudinal wound diameter than the control group on the 3^rd^ day after wounding (Fig. 2B, arrows), corroborating the macroscopic analysis. Although the total healing time was not affected by EPA, at 21 days after wounding, animals that received EPA-rich oil presented packed parallel layers of collagen, whereas in the control mice there was a basket-weave organization of collagen bundles (Fig. 2C). Moreover, qualitative analysis showed that there were more hair follicles on control skin than in the EPA group (Fig. 2C) indicating a delay in the return of skin function in EPA mice. Control group showed thick collagen fiber deposition and fasciculate orientation (detail), thin squamous stratified epithelium and bulbs of hair follicles and sebaceous glands at the lesion area. EPA group showed impaired thick collagen fiber deposition and mixed orientation (detail), thicker squamous stratified epithelium and scarce presence of bulbs of hair follicles and sebaceous glands at the lesion area (Fig. 2C).

As progression of remodeling of the wound site occurs, we observed increase in skin appendages, such as hair follicles and sweat glands. In addition, the parallel organization become a reticular organization like a “basket weave”^20^.

Taken together, these results indicate that EPA-rich oil supplementation impaired the wound healing process, retarding collagen organization. Considering that the inflammatory phase influences the next phases, we investigated the effects of EPA supplementation on the inflammatory phase of wound healing.

### EPA-rich oil modulated skin immunophenotypes and cytokines after wounding

In wound healing studies it is important to characterize cellular responses; therefore, flow cytometry was performed to determine the phenotype of the cellular populations in wound tissue and Enzyme Linked Immunosorbent Assays (ELISA) were performed to determine the cytokine profile in wound tissue, during the wound healing process.

To characterize the cells, present in the skin before wounding (unwounded skin), or during the inflammatory phase (3^rd^ day after wounding) and the proliferative phase (10 days), markers for neutrophils (CD45^+^Ly6G^+^), M1 macrophages (CD45^+^F4/80^+^CD11c^+^), M2 macrophages (CD45^+^F4/80^+^CD206^+^), T helper lymphocytes (CD45^+^TCRb^+^CD4^+^) and T cytotoxic lymphocytes (CD45^+^TCRb^+^CD8^+^) were used. The gating strategy is shown in Supplemental Fig. 2A,B.

**Figure 3:**
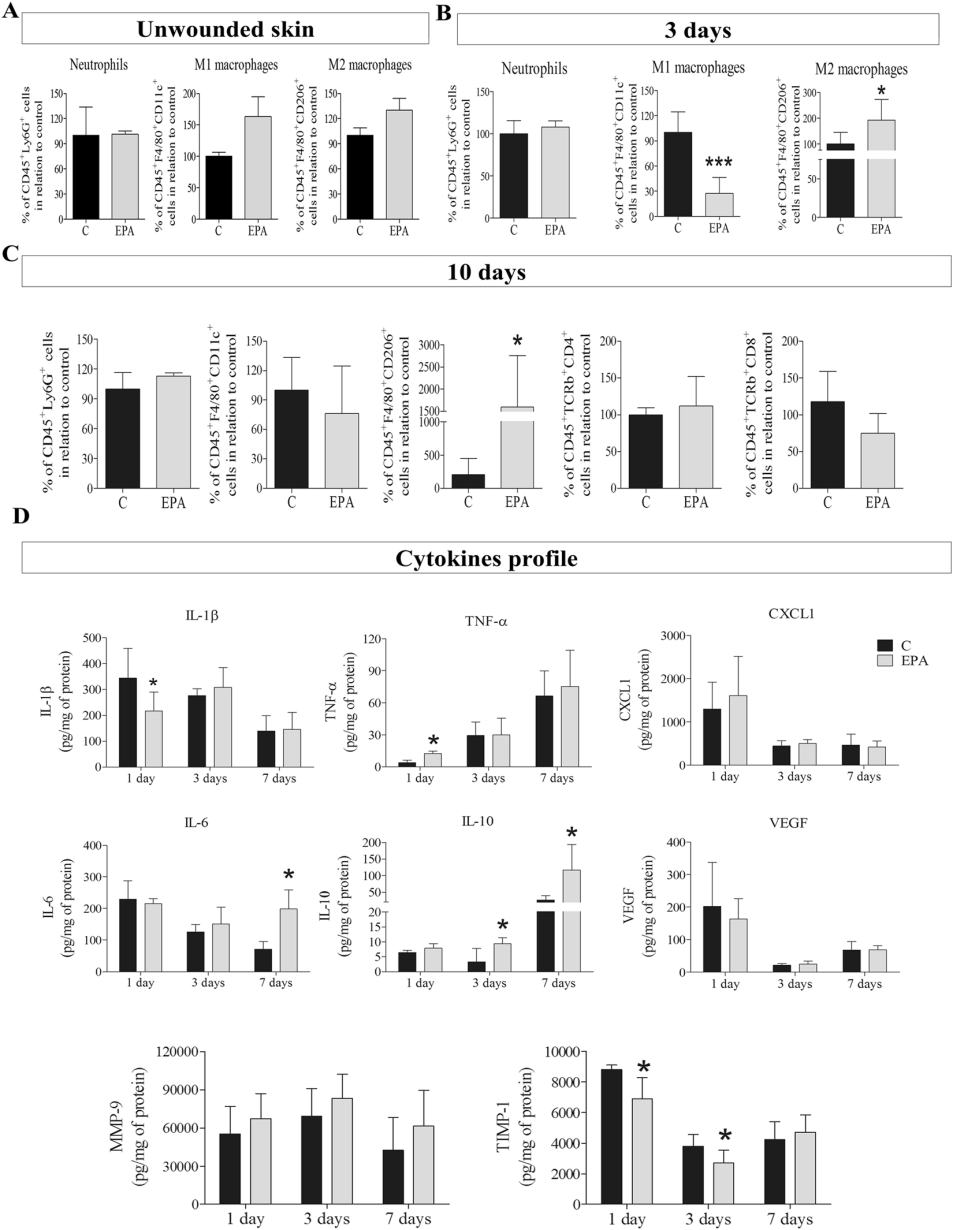
Immunophenotyping and cytokine profile of wound tissue in the control group (C, Black bar) and EPA-group (EPA, grey bar). (**A**–**C**) Percentage of positive neutrophils (CD45^+^Ly6G^+^), M1 macrophages (CD45^+^F4/80^+^CD11c^+^), M2 macrophages (CD45^+^F4/80^+^CD206^+^), T helper lymphocytes (CD45^+^TCRb^+^CD4^+^) and T cytotoxic lymphocytes (CD45^+^TCRb^+^CD8^+^) were quantified by flow cytometry in scar tissue harvested: (**A**) before skin lesion (unwounded); (**B**) 3 days after wounding and; (**C**) 10 days after wounding. (**D**) Concentrations of interleukin 1-β (IL- β), tumor necrosis factor-α (TNF-α), keratinocyte chemoattractant (CXCL1), interleukin-6 (IL-6), interleukin-10 (IL-10), vascular endothelial growth factor (*VEGF*), metalloproteinase-9 (MMP-9) and tissue inhbitor of metalloproteinase-1 (TIMP-1) in wound tissue was analyzed by ELISA in tissue collected 1, 3 and 7 days after wounding (n = 5–12 animals/group). Values are expressed as mean ± SD. *p < 0.05; **p < 0.01, ***p < 0.001 indicates significant differences in relation to control as indicated by *t* Test and Mann Whitney posttest.

EPA-rich oil did not alter the percentages of neutrophils or macrophages (M1 and M2) (Fig. 3A), or the concentrations of tumor necrosis factor-α (TNF-α) and macrophage inflammatory protein-2 (MIP-2/CXCL-2) in unwounded skin (data not shown), indicating no disturbance in skin homeostasis. Interleukin-1β (IL-1β), keratinocyte chemoattractant (KC/CXCL-1), interleukin-6 (IL-6) and interleukin-10 (IL-10) concentrations were not detected in unwounded skin (data not shown).

The oral administration of EPA-rich oil decreased the percentage of CD45^+^F4/80^+^CD11c^+^ cells (3^rd^ day) and increased the percentage of CD45^+^F4/80^+^CD206^+^ cells in scar tissue at the 3^rd^ and 10^th^ days after wounding (Fig. 3B,C). These results suggest an anti-inflammatory effect of EPA during the tissue repair. No alterations were observed in percentage of CD45^+^TCRb^+^CD4^+^ and CD45^+^TCRb^+^CD8^+^ cells (Fig. 3C).

Then we analyzed the cytokine profile through the wound healing process. EPA reduced the concentrations of IL-1β and increased TNF-α 1 day after skin wounding (Fig. 3D). On the other hand, EPA increased IL-10 concentrations at the 3^rd^ day until the 7^th^ after wounding. There was also an increase in IL-6 concentrations at this time point in scar tissue (Fig. 3D). We observed no alterations in MMP9 concentrations at the times analyzed. However, supplementation with EPA-rich oil decreased TIMP-1 levels on the 1^st^ and 3^rd^ days after lesion (Fig. 3D).

Take into account the elevated percentages of macrophages on wound tissue at the 3^rd^ day after wound induction, we isolated peritoneal macrophages after inoculation with thioglycolate and evaluated the cytokine production after 24 hours in cell supernatant. The production of IL-1β, CXCL-1, IL-6 was not altered in macrophages isolated from the EPA-mice at any condition studied (Supplemental Fig. 3B). On the other hand, macrophages isolated from the EPA-mice reduced TNF-α and increased IL-10 production after LPS stimulated (Supplemental Fig. 3B).

Considering these findings, we hypothesized that the deleterious effects of oral administration of EPA-rich oil on wound closure and collagen organization could be due to IL-10 induction, since this cytokine, which is produced by M2 macrophages, was increased during the process. To test this hypothesis, we repeated the analysis in IL-10^−/−^ mice supplemented with EPA-rich oil.

### Absence of IL-10 abolished the effects of EPA on wound healing

IL-10^−/−^ mice were orally supplemented daily with 2 g of EPA-rich oil/kg body weight during 4 weeks. After this period, a wound of 1 cm^2^ was surgically induced in their dorsum and the wound closure was monitored until the 21^st^ day. No alterations were observed in nutritional parameters (data not shown).

**Figure 4:**
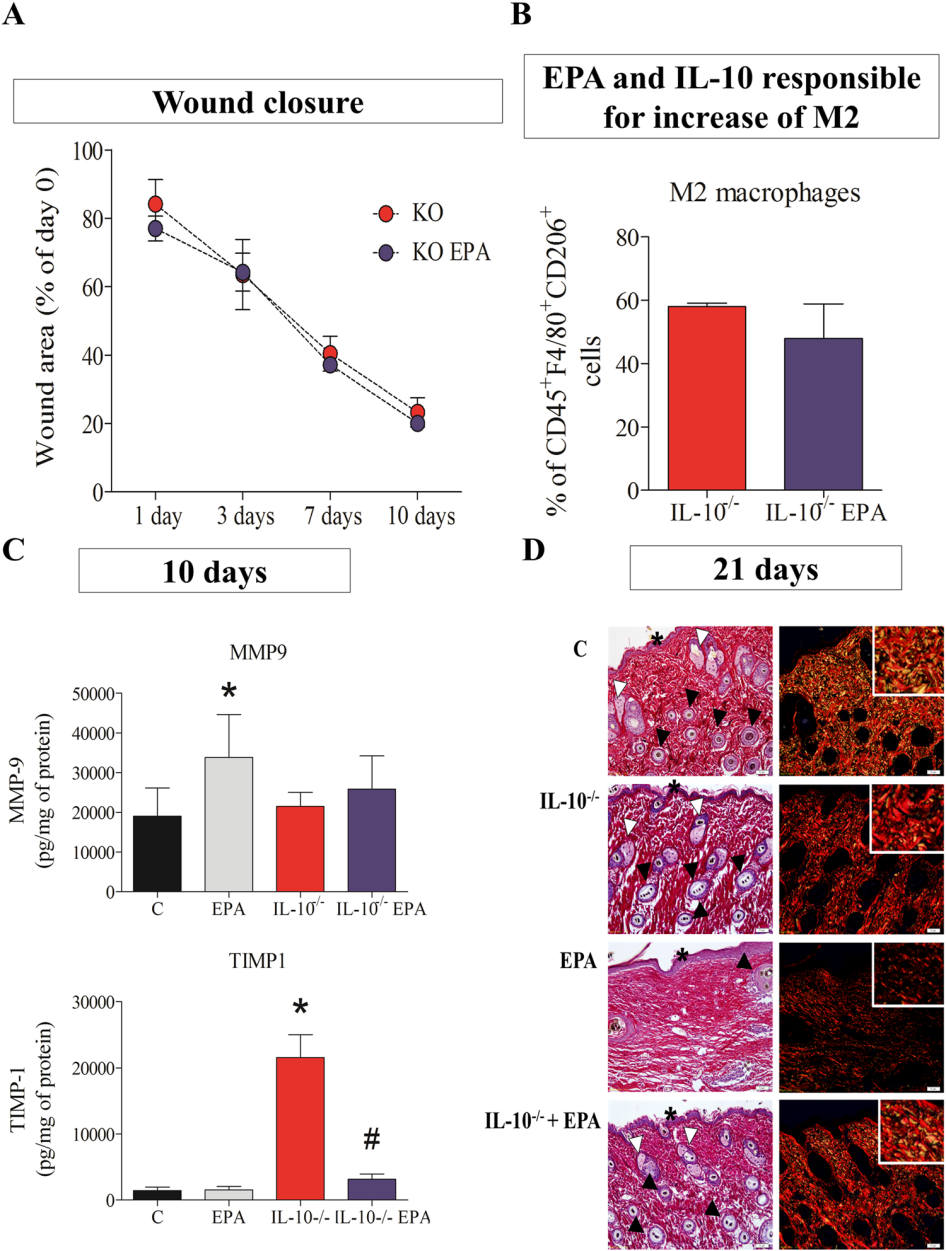
Wound healing, immunophenotyping, MMP9 and TIMP-1 production of scar tissue on 10^th^ day after wounding in IL-10^−/−^ mice and IL-10^−/−^ supplemented daily with EPA-rich oil. (**A**) Wound area percentages during the experimental period in IL-10^−/−^ mice (blue line) and IL-10^−/−^ mice supplemented daily with EPA-rich oil (red line) (n = 5–7 animals/group). Values are expressed as mean ± SEM. The comparison between the groups was made through two-way analysis of variance (ANOVA) and Bonferroni post-test. (**B**) Percentage of positive M2 macrophages (CD45^+^F4/80^+^CD206^+^), were quantified by flow cytometry in scar tissue harvested 10 days after wound induction. Values are expressed as mean ± SD (n = 3–4 animals/group). The comparison between the groups was made through *t* Test and Mann Whitney posttest. (**C**) MMP9 and TIMP-1 quantification of scar tissue collected 10 days after wound induction. Values are expressed as mean ± SD (n = 5–8 animals/group). *p < 0.05 indicates significant differences in relation to control and ^#^p < 0.05 indicates significant differences in relation to IL-10^−/−^. (**D**) Representative photomicrographs of skin stained with picrosirius and hematoxylin. The examination without (left) and with (right) polarized light revealed the organization and heterogeneity of collagen fiber orientation, squamous stratified epithelium (asterisk) and bulbs of the hair follicles (black arrowhead) and sebaceous glands (white arrowhead) in 21^st^ day after lesion induction (n = 2–5 animals/group). Scale bar = 50 µm.

When we compared IL-10^−/−^ mice and IL-10^−/−^ mice supplemented with EPA-rich oil, the deleterious effect of EPA on wound closure was abolished (Fig. 4A).

The M2 population of the wound at the 10^th^ day was analyzed by flow cytometry and we observed no difference between the IL-10^−/−^ mice and IL-10^−/−^ mice treated with EPA-rich oil (Fig. 4B). These results suggest that the increase of M2 in the animals supplemented with EPA may be related to the increase of IL-10 at 3 and 7 days after tissue injury.

At this time point there was also an increase of MMP9 in the EPA-group in comparison to the Control group (Fig. 4C) and an increase in TIMP-1 in IL-10^−/−^ mice in comparison to Control group (Fig. 4C). On the other hand, there is a reduction of TIMP-1 in the IL10^−/−^ mice treated with EPA-rich oil in comparison to IL10^−/−^ mice (Fig. 4C).

Only the EPA group showed impaired thick collagen fiber deposition and orientation, thicker squamous stratified epithelium and scarce presence of bulbs of hair follicles and sebaceous glands at the lesion area (Fig. 4D).

Considering the results presented in the Figs 3 and 4, the supplementation with EPA-rich oil disturbed the MMP-9 and TIMP-1 balance, impairing the collagen organization. This effect seems to be related with IL-10, since the IL-10^−/−^ mice supplemented with EPA-rich oil reestablished the collagen organization and no alterations in MMP-9 concentrations were observed.

## Discussion

Polyunsaturated fatty acids (PUFAs) of the ω-3 family have been recognized as important anti-inflammatory agents^16^, reducing the risk of cardiovascular disease, cancer, Alzheimer’s disease and having a protective effect in rheumatoid arthritis, asthma, Crohn’s disease and psoriasis^21^. However, their effects on skin wound healing are controversial.

**Figure 5:**
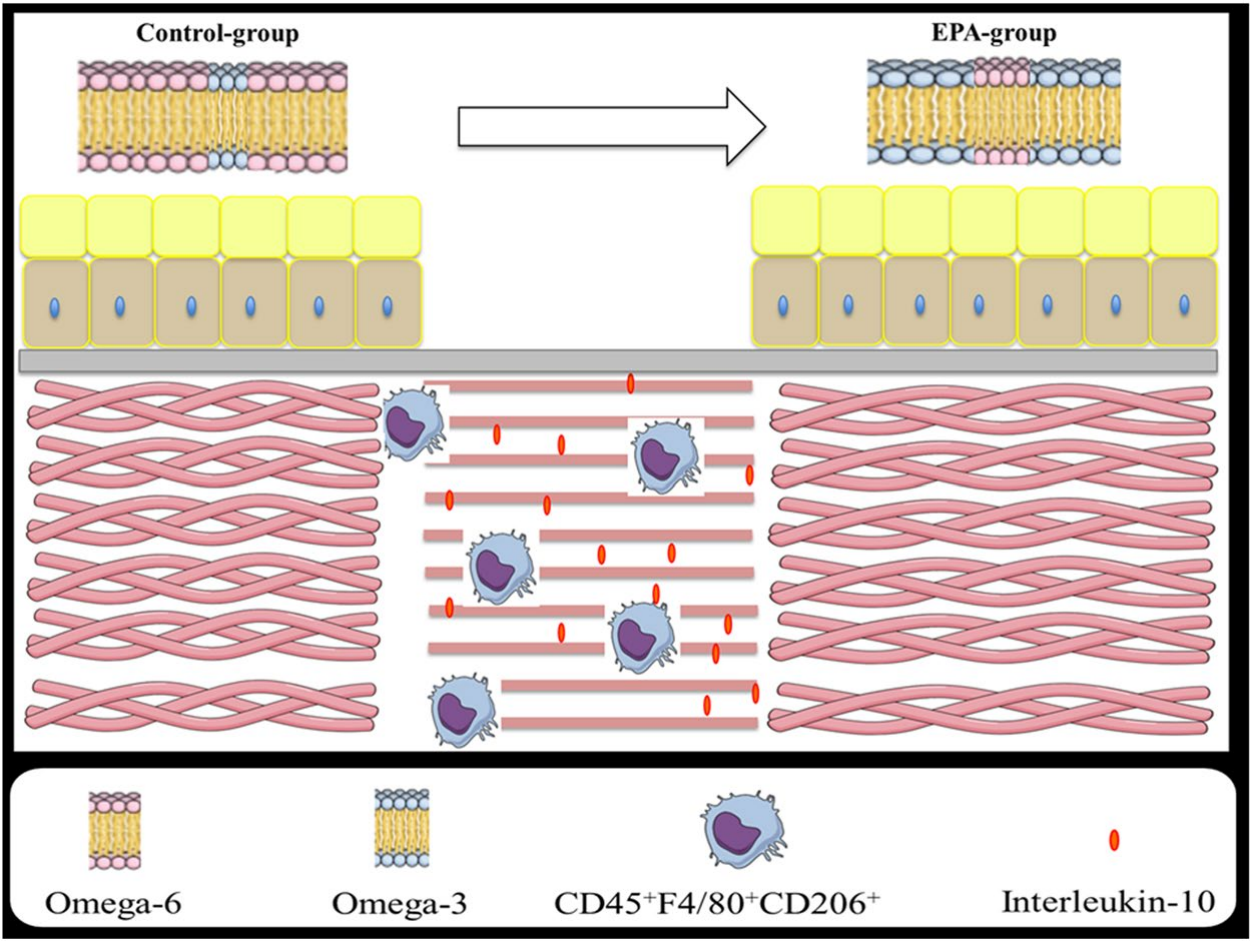
Effects of EPA on the wound healing process. The intake of EPA-enriched oil leads to an increment in the incorporation of omega-3 in cell membranes, an increase of M2 macrophages and an increase of a key anti-inflammatory cytokine produced by this cell population, interleukin-10, in the scar tissue. This anti-inflammatory effect of EPA is associated with delayed of wound closure and affects the reorganization of collagen.

In the EPA-group, the intake of EPA-enriched oil leads to an increment in the incorporation of omega-3 in cell membranes in comparison to the Control group. This increase was related with elevation in M2 macrophages and in interleukin-10, a key anti-inflammatory cytokine produced by this cell population. This anti-inflammatory effect of EPA was associated with delayed wound closure and reorganization of collagen because absence of IL-10 abolished the deleterious effects of EPA and the increase of M2 macrophages (Fig. 5).

There are few studies showing the effects of ω-3 fatty acids on wound healing and the conclusions are inconsistent. In beagle dogs fed with a menhaden oil rich diet (the ω-6/ω-3 ratio was 0.3 compared with a control diet with the ω-6/ω-3 ratio of 7.7), there was a reduction in epithelialization and contraction of the wounds^22^. These results were related with a tendency to reduce prostaglandin E_2_ (PGE_2_) concentrations as well as inhibition of tissue perfusion^22^. On the other hand, in Sprague-Dawley rats, lipid emulsion administration improved the wound healing process due to a reduction in swelling around the wound in the early stages and increase of new vessel formation on granulation tissue in the proliferative phase^23^. However, the lipid emulsion used was composed by 30% of soybean oil, 30% of medium-chain triglycerides, 25% of olive oil and 15% of fish oil^23^. Thus, the effects observed in wound healing can be due to the mixture of oils, since we and others already demonstrated that omega-6 fatty acids, found in soybean oil, can improve the healing process^8^. Enteral administration of formulas enriched with arginine and ω-3 fatty acids reduced the number of patients with wound healing complications after undergoing gastric surgery^24^. In this study, the formulation with ω-3 fatty acids contained other nutrients such as ribonucleic acid (RNA) and arginine, that may influence the tissue repair process. On the other hand, healthy male and female volunteers (ages 18–45 years) that were supplemented with EPA (1.6 g) and DHA (1.1 g) daily during 4 weeks and submitted to blister wounds, had increased IL-1β concentrations in the blister fluid and had prolonged healing time, when compared to the placebo group that received mineral oil^25^.

Thus, results seem to vary according to the experimental models, doses and methodologies used.

In the present study, EPA-rich oil administration modified the serum concentrations of fatty acids, and consequently, their incorporation into skin. There was also a reduction of ω-6/ω-3 ratio in serum and skin on EPA-group (Fig. 1).

Menhaden oil enriched-diet enhanced EPA and DHA in mouse skin and serum within 2 weeks. This increase plateaued after 4 weeks of supplementation^26^. Although the reference range of fatty acids is important to evaluation and interpretation of pharmaceutical or dietary intervention, “normal” levels of circulating and tissue fatty acids are not defined^27^.

Considering the data obtained by gas chromatography, the supplementation protocol used was able to alter the fatty acids composition of mice serum and skin. These results are important since the membrane lipid composition play a key role in cutaneous inflammation because it regulates the immune response^28^.

After 3 days of skin lesion, macrophages are the predominant immune cells found at the wound site. In the wound, macrophages remove dead cells and secrete many cytokines and growth factors that regulate the proliferative phase to stimulate migration, proliferation and differentiation of fibroblasts, keratinocytes and endothelial cells^29^. In the remodeling phase, macrophages can release enzymes that alter the composition of the extracellular matrix (ECM) and the structure of the wound bed^29^.

The apparent discrepancy between the increase in TNF-α concentrations (at the 1st day) and the elevation in M2 macrophages (CD45^+^F4/80^+^CD206^+^) at the 3^rd^ day after wound induction can be explained by the color wheel macrophage activation model, as proposed by Mosser *et al*.^30^. In this model, macrophage phenotypes are designated as primary colors (red, yellow and blue). The interconnection among the primary colors generates the secondary colors such as green. So, secondly in this model the green color represents macrophages that share functions of wound healing and regulatory macrophages, for example^30^. More recently, Hu *et al*. (2017) described 5 different macrophage phenotypes (M1, M2a, M2b, M2c and M2d) during the wound healing process, based on single cell RNAseq analysis^31^. However, the functional significance of all these phenotypes is not fully understood yet. Both these publications show the complexity of macrophage polarization during the wound healing process.

Literature is scarce in relation to the effects of EPA on macrophage polarization. *Fat-1* transgenic mice (animals that endogenously produce ω-3 fatty acids from ω-6) fed a 60% calorie high-fat diet (HFD) during 10 weeks showed reduced recruitment of pro-inflammatory M1 and increased anti-inflammatory M2 macrophages to adipose tissue^32^. These effects were associated with suppression of multiple kinases, such as IkB kinase, AKT, and focal adhesion kinase^32^.

In the present study, EPA-group decreased the percentage of CD45^+^F4/80^+^CD11c^+^ cells and increased the percentage of CD45^+^F4/80^+^CD206^+^ cells on the 3^rd^ day after wounding (Fig. 3B).

After arrival at the wound site, macrophages augment the production of pro-inflammatory mediators, such as cytokines, to amplify the inflammatory response^7^. Cytokines are soluble proteins that modulate growth, differentiation and metabolism of target cells^33^.

The elevation in IL-6 concentrations at the 7^th^ day may be related to the increase of M2-macrophages, since IL-6 has a pleiotropic action. IL-6 has a dual function in the immune system: it exerts a pro-inflammatory or anti-inflammatory effect depending on the microenvironment^34^. Although IL-6 is commonly associated with proinflammatory functions and is implicated in the pathogenesis/pathophysiology of numerous inflammatory diseases, it may potentiate the polarization of alternatively activated macrophages based on increased expression of markers: arginase-1, Ym1, and CD206, through IL-4 and IL-13 stimulation^35^. In the study by FU *et al*., (2017), a positive correlation was demonstrated between the number of M2 macrophages (CD163^+^CD206^+^) and IL-6 production. This effect may be due to the greater activation of STAT3^34^. In another study, it was demonstrated that, after infection by *Trypanosoma cruzi*, IL-6 boosted the recruitment of monocytes and determined the profile of M2 cardiac macrophages during infection^36^.

However, it is unclear what the effects of ω-3 fatty acids on wound cytokine concentrations are. Most of the studies that investigated the effects of ω-3 fatty acids on cytokine production have focused on cell culture and not in whole tissue^37–39^. So, in all these studies, the cells analyzed have never been in contact with the wound environment.

*In vitro* treatment of monocytes with EPA or DHA (25–100 μM) during 19 hours did not alter the production of TNF-α and IL-6^40^. On the other side, T lymphocytes reduced in dose-dependent manner the production of TNF-α after EPA or DHA treatment^40^. Considering that in a wound there are different cell types, in different stage of activation (M1 and M2 for example), as demonstrated in the present study, the interpretation of cytokines concentrations is much more complex than in a cell culture study. It is know that microenvironment influences the biology of the entire tissue^38^. It doesn’t mean that our results are not comparable with the literature; it just indicates that the comparisons are not direct.

Considering the significant increase in IL-10 concentrations during the wound healing process in the EPA group and the effects of this cytokine on the inflammatory response, we hypothesized that IL-10 could have a central role in the effects of EPA on wound healing. This hypothesis was confirmed since when IL-10^−/−^ mice were treated with EPA-rich oil, the healing time was normalized and the collagen organization was improved (Fig. 4). These observations are suggestive that decreased synthesis and/or altered molecular assembly of ECM components in the EPA-group may be due to the increase in IL-10.

Other groups already demonstrated the deleterious effects of IL-10 on wound healing. In 2007, EMING *et al*. demonstrated for the first time that IL-10 can delay tissue repair. The authors observed that mice deficient in IL-10 presented an accelerated tissue repair when compared to WT mice^41^. At the same time, the elevation in IL-10 concentrations at wound tissue is related with poor wound healing since it impairs the infiltration inflammatory cells to the injured area disrupting the entire process as demonstrated by KIMURA *et al*.^42^.

The effects of IL-10 on wound healing process are dose-dependent, as demonstrated by Gordon *et al*. (2008). In this study, the adenoviral-mediated overexpression of IL-10 prevented scar formation. In a titer experiment, it was demonstrated that the ideal IL-10 concentration achieved to improve the healing was 831, 4 pg/ml^43^. This means that IL-10 concentrations above or below this value compromises the healing process. Considering that, in our study, the highest IL-10 concentration, measured at wound site, was ± 116 pg/mg of protein, it means a 8-fold less IL-10 in our work than in Gordon’s study.

*In vitro* studies demonstrated that IL-10 directly regulates synthesis and degradation of various extracellular matrix molecules by different fibroblastic cell types. It was demonstrated that IL-10 down-regulates mRNA expression of collagen I and fibronectin, but up-regulates decorin and collagenase-1 in a time- and dose-dependent manner in human skin fibroblasts^44^. These effects seem to be related to the inhibition of TGF-β1 pathway^44^.

There are few studies that investigate the effects of omega-3 fatty acids on collagen biology. It is know that peroxisome proliferator-activated receptor (PPAR-γ) regulates numerous biological processes, among them, collagen synthesis^45^. An *in vitro* study, using mouse embryonic fibroblasts (MEF) demonstrated that loss of PPAR-γ is associated with up regulation of collagen synthesis, at least in part, due to the TGF-β pathway^45^. Omega-3 fatty acids, such as EPA, are natural ligands of PPAR-γ^46–48^. So, one possibility for the results observed in the present study is that EPA activated PPAR-γ which then disturbed collagen organization. This hypothesis is in agreement with the observations that, *in vitro* treatment of keloids fibroblasts with DHA (0–100 μM) reduced α-smooth muscle actin, type III collagen and TGF-β1 receptor expressions. Once again, these effects seem to be related to PPAR-γ signaling^49^.

Metalloproteinase-9 (MMP-9) is a type IV collagenase found at elevated levels in chronic wounds. As wounds heal, MMP-9 diminishes^50^. Thus, the increase of MMP9 10 days after wound healing can explain the delayed collagen organization in the EPA group (Fig. 4C). The increase in TIMP in IL-10^−/−^ mice could be related with better collagen organization, since TIMP-1 inhibits MMP-9. Although there was a reduction in TIMP-1 in the IL-10^−/−^ mice treated with EPA-rich oil, the Sirius Red analysis showed a better collagen organization.

In the present study, more sebaceous glands and hair follicles were observed in IL-10^−/−^ mice fed EPA (Fig. 4D). The presence of these appendages can improve the wound healing process, considering that, in the epithelial compartment of the folicles, there are stem cells that can differentiate into numerous cell types^51^. Furthermore, hair folicles are a source of pro-angiogenic factors, such as VEGF^52^. Few studies have investigated the effects of fatty acids on skin hair growth^53,54^.

Taken together, our results demonstrate that EPA had a predominantly anti-inflammatory character, demonstrated by elevation in CD45^+^F4/80^+^CD206^+^ cells and the increase in IL-10 concentrations in scar tissue. IL-10 seems to have a deleterious effect on wound healing and on collagen organization in healthy animals. Collectively, findings from this study enhance our understanding of EPA-rich oil effects on wound healing.

## Materials and Methods

### Ethical approval

Mice were maintained according to the National Institute of Health guidelines for the use of experimental animals with the approval of the Care of Animals and Ethical Committee for Animal Research of the Institute of Biology/University of Campinas.

### Animals

Eight-week-old C57Bl6 male mice were purchased from the Animal Breeding Center of University of Campinas (Campinas, Brazil). IL-10^−/−^ mice were purchased from the Animal Facility of the School of Medicine of Ribeirão Preto/University of São Paulo (Ribeirão Preto, Brazil). All mice were maintained at 23 ± 2 °C under a 12:12 h light/dark cycle with access to water *ad libitum*.

Animals received chow (Nuvital, Curitiba, Brazil) containing 22% protein, 4.5% fat, 40.8% carbohydrate, 8% fiber, reaching 3.0 kcal/g total metabolizable energy. The fat component of chow contains 1.8% of EPA; 42.6% of linoleic acid; 17.2% of oleic acid; with a total of 35.4% of saturated fatty acids and 64.6% of unsaturated fatty acids^55^.

### Administration of EPA-rich oil

EPA-rich oil at a dose of 2 g/kg body weight (bw)^56^ was administered by gavage daily during four weeks (Supplemental Fig. 1A). Considering the fatty acid composition of the chow diet (RODRIGUES *et al*., 2010) and the food ingestion of the animals (4 g/day- Supplemental Fig. 1B), the amount of EPA-rich oil administered represents an 2-fold increase in the amount of omega-3 fatty acid ingested by mice consuming AIN-93M commercial chow^57^.

Control animals received 2 g/kg body weight of water by gavage. Having in mind that the caloric content of the fatty acids provided in the oil was small (1.98 kcal/day), we chose to use water as an inert liquid control^11^. This approach did not alter nutritional parameters (food ingestion, water ingestion and weight gain) (Supplemental Fig. 1B).

In the current study, the decision to use the chosen EPA-rich oil dose was based on a dose-response experiment previously performed (data not shown).

The EPA-rich oil was donated by Naturalis®. Analysis of the composition of EPA-rich oil was conducted by the University of São Paulo, Faculty of Pharmaceutical Sciences, Department of Food and Experimental Nutrition^58^. The results showed that the oil used contains 60.9% EPA, 16.3% DHA, 1.6% oleic acid, among others. Polyunsaturated fatty acids constitute 95.9%, monounsaturated fatty acids 2.5% and saturated fatty acids 0.8% of the oil^58^.

### Wound induction

After 4 weeks of EPA-rich oil supplementation, animals were anesthetized with 30 µL xylazine and ketamine solution and an area of 1 cm^2^ of skin in the dorsal region was removed by surgery. Animals were sacrificed 1, 3, 7, 10 and 21 days after the surgery by inhalation with isoflurane (12%).

### Serum collection and determination of fatty acids composition

Blood was collected at 3, 7 and 21 days after the surgery: after thoracotomy, the blood was collected by cardiac puncture from the left ventricle. Blood was centrifuged at 3000 rpm for 15 minutes at 4 °C. The serum was used for fatty acid determinations. Total lipid was extracted from serum and from skin into chloroform:methanol (2:1 vol/vol) as were described^59^. Lipids from skin tissues were separated into the major fractions phosphatidylcholine (PC) and phosphatidylethanolamine (PE) by solid-phase extraction on aminopropylsilica cartridges (Sep Pak C18 Cartridges, Waters^®^, Milford, Massachusetts, EUA). Fatty acid methyl esters (FAMEs) were formed by incubation of lipids with methanol containing 2% (vol/vol) H_2_SO_4_ at 50 °C for 2 hr. After allowing the tubes to cool, samples were neutralized with a solution of 0.25 M KHCO_3_ and 0.5 M K_2_CO_3_. FAMEs were extracted into hexane, dried down, dissolved in a small volume of hexane and analyzed by gas chromatography (Hewlett-Packard 6890 chromatograph (Hewlett-Packard,California, United States), as were described^59^. For details of protocol, please see the Supplemental Information.

The omega-6/omega-3 ratio was obtained by performing the sum of the five omega-6 fatty acids in each group and the sum of the five omega-3 fatty acids in each group. Then, we divided the omega-6 per omega-3 value of each group separately.

### Histological analysis

Skin samples were fixed in formaldehyde 4% diluted in 0.1M phosphate-buffered saline (PBS; pH 7.4) for 24 h at 4 °C. The tissues were dehydrated in graded concentrations of alcohol, embedded in paraffin and sectioned transversely at a width of 5 µm. Serial sections were mounted on slides and stained with hematoxylin/eosin for H&E analysis or Sirius Red using hematoxylin as previously described^60^.

Wound re-epithelialization was measured by morphometric analysis of wound sections. Sections taken from the center of the wound were stained with H&E and the distance between the wound edges, defined by the distance between the first hair follicle encountered at each end of the wound, and the distance that the epithelium had traversed into the wound, were measured using image analysis software^61^.

The collagen fiber organization was detected by Sirius Red staining combined with polarized light detection^62^. Briefly, the slides were incubated with Sirius Red solution dissolved in aqueous saturated picric acid for 1 h, washed in tap water, incubated for 15 min in hematoxylin, dehydrated and mounted. The sections were then examined and imaged using a Leica stereoscopic microscope (MZ10F, Wetzlar, Alemanha) coupled with a Leica camera (DFC310 FX, Wetzlar, Alemanha) and Olympus microscope (U-LH100HG, Shinjuku, Tokyo, Japan) with images representative of the histological structures registered in digital image capture and analysis system (Camera: Olympus/U-TVO.63XC/T2, Shinjuku, Tokyo, Japan).

### Wound measurement

To evaluate wound closure, the wounds were photographed daily with a Sony® cyber shot (model DSC-S950S 10MP 4_Optical zoom) by the same examiner at the moment of wound induction and 1, 3, 7, 10 and 16 days after wounding. After digitalization, the wound area was measured using *ImageJ* software® (National Institutes of Health, Bethesda, MD). Wound closure was defined as a reduction of wound area and results were expressed as percentage (%) of the original wound area^63^.

### Phenotypic characterization of leukocytes by flow cytometry

The expression of CD45, Ly6G, F4/80, CD11c, CD206, TCRb, CD4 and CD8 in the wound was evaluated by flow cytometry. The tissue collected at the moment of wound induction was denominated as Unwounded skin. Moreover, scar tissue from mice was collected in the different periods (3 and 10 days). After collection, tissues were washed twice in PBS, cleaved with scissors and dissociated by enzymatic digestion (40 U/mL of collagenase IV and 40 µg/mL of DNAse).

The resulting cell suspension (1 × 10^6^ cells) was washed twice with PBS containing 1% albumin and resuspended in 100 μl of PBS. CD45-FITC, Ly6G-PE, F4/80-APC-Cy7, CD11c-PE, CD206-APC, TCRb-PE-Cy7, CD4-APC, CD8-PE conjugated specific antibody was added to this suspension (1:10) and the cells were incubated at 4 °C for 15 min protected from light. Negative control cells were incubated with the non-reactive labeled IgG antibody. After this period, the cells were washed twice with PBS and analyzed on the BD-FACS Accuri flow cytometry® (BD Bioscience, Maryland, USA), and the fluorescence was determined by the specific filters for each fluorochrome. One hundred thousand events were acquired per sample in histograms. Histograms were analyzed using BD Accuri software (BD Bioscience, Maryland, USA). The gate strategy is shown in Supplemental Fig. 2A.

Due to the variations that occurred between the three independent experiments, we chose to normalize the percentages by the mean of the control group of each day of experiment.

### Determination of cytokine concentrations in wound tissue

Wound tissues removed at 0 hours, 1, 3 and 7 days after wound induction were immediately packed in dry ice and kept frozen (−80 °C) until they were homogenized. For homogenization, phosphate-buffered saline was supplemented with protease inhibitor cocktail tablets (Roche Diagnostics®, Mannheim, Germany). Tissue (100 mg) was homogenized with a Polytron PT 1200 (Kinematica®, Lucerne, Switzerland). Samples were then sonicated for 1 minute and centrifuged at 5,000 rpm at 4 °C for 10 min^63^. The concentrations of cytokines (IL-1β, TNF-α, CXCL-1, IL-6 and IL-10), growth factors (VEGF), collagenase matrix metalloproteinase-9 (MMP9) and the tissue inhibitor of metalloproteinase (TIMP-1), in the supernatants, were assessed by ELISA using the Duo Set kit (R&D System®, Minneapolis, MN, USA). The concentrations were normalized by the amount of protein in the samples, determined by the method were described^64^.

### Genotyping of IL-10^−/−^ mice

DNA was extracted from tail and mice were genotyped by electrophoresis of the generated compounds submitted to polymerase chain reaction (PCR). The DNA extraction and the PCR were performed using the REDExtract-N-Amp^TM^ Tissue PCR Kit (Sigma®, St. Louis, Missouri, EUA). Mice that presented a band around 200 bp correspond to the wild type pattern (C57BL/6), while a band around 400 bp corresponds to the IL-10^−/−^ (KO) genotype (Supplemental Fig. 4).

For PCR the nucleotide sequences were:

IMR 86 5′-GTGGGTGCAGTTATTGTCTTCCCG-3′;

IMR 87 5′-GCCTTCAGTATAAAAGGGGGACC-3′

IMR 88 5′-CTTGCGTGCAATCCATCTTG-3′

### Statistical analysis

Statistical analyses were performed using GraphPad Prism 5 (GraphPad, San Diego, CA). Significance of difference was analyzed using Two-way ANOVA was used in Figs 1A,C,E; 2A and 4A; Supplementary Fig. 1B. On the other hand, we used Student’s t test to analyze the data shown in Figs 1B,D,F; 2B; 3A–D and 4B. We used One-way ANOVA for data shown in Fig. 4C. All data are presented as mean ± SD and p < 0.05 was considered significant.

## Supplementary information

Supplementary information
