# Supplementary material for: Oral administration of EPA-rich oil impairs collagen reorganization due to elevated production of IL-10 during skin wound healing in mice
Source: Sci Rep. 2019 Jun 24;9:9119. doi: 10.1038/s41598-019-45508-1 (PMC6591225; doi:10.1038/s41598-019-45508-1)
Supplement: Supplementary file 1 — Supplementary information [file 41598_2019_45508_MOESM1_ESM.docx]

**Supplementary Figures**

**
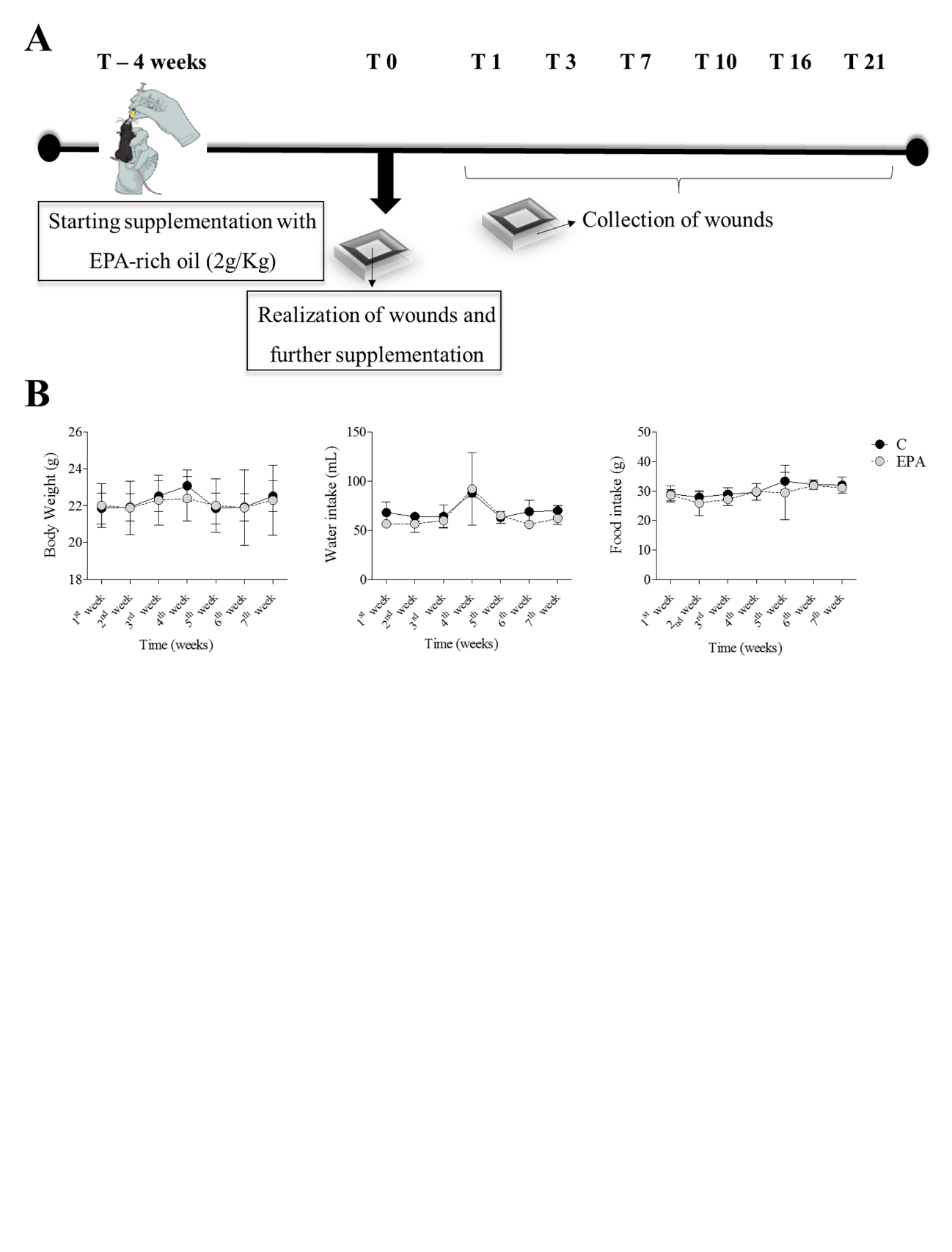
**

**Supplemental Figure 1.** Design of experiments and nutritional parameters. **(A)** Mice were supplemented daily for 30 days with EPA-rich oil at a dose of 2 g/kg of bw, the wound was induced by a surgery and the wound was collected after 1, 3, 7, 10 and 21 days after wounding for analysis. **(B)** Nutritional parameters: body weight (bw), food intake and water intake (n=4-10 animals/group). Values are expressed as mean. The comparison between the groups was made through two-way analysis of variance (ANOVA) and Bonferroni post-test.


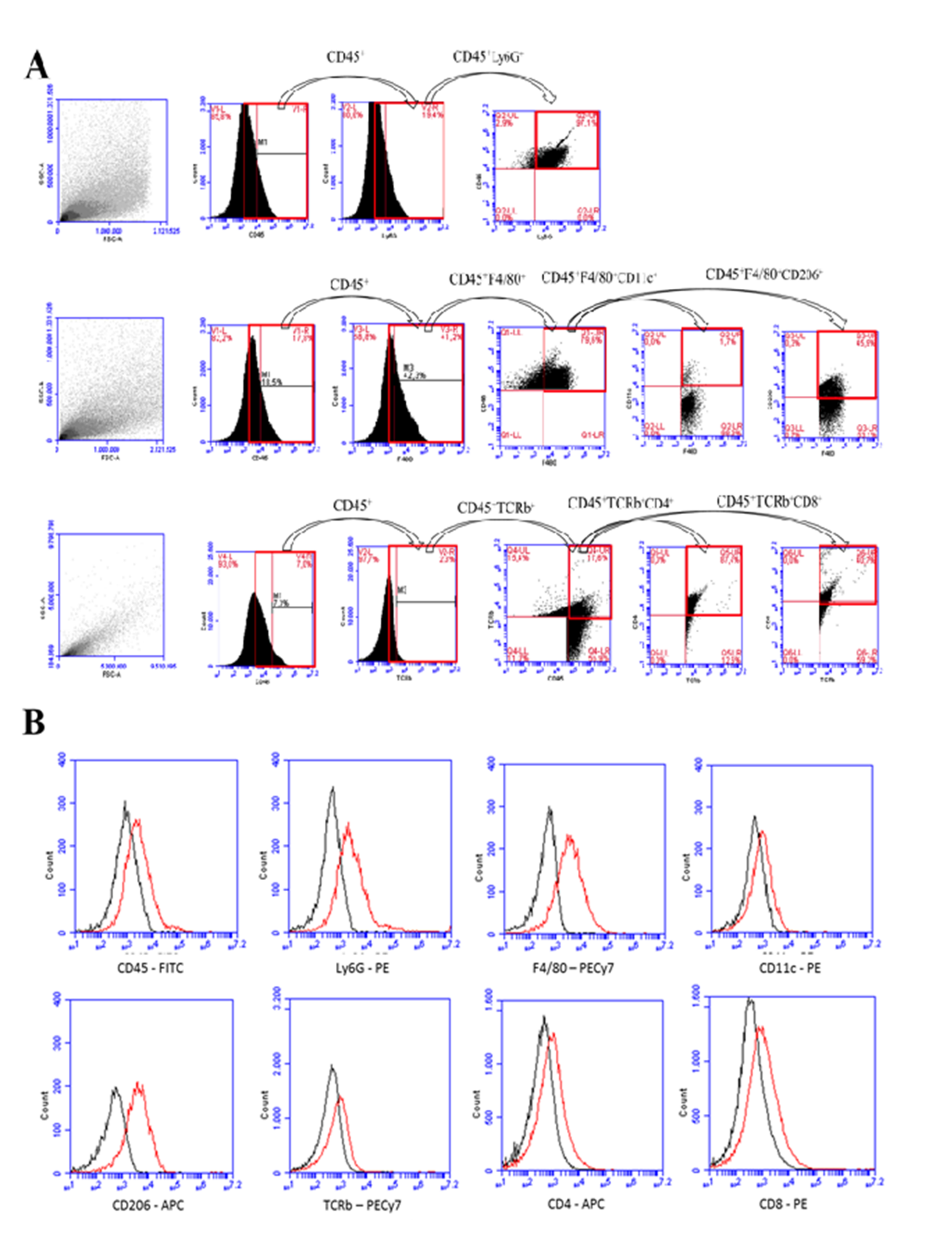


**Supplemental Figure 2.**  **Flow cytometry experiments. (A)** The gating strategy. **(B)** functionality of antibodies. Black: unmarked cells. Red: Cells marked with antibody.


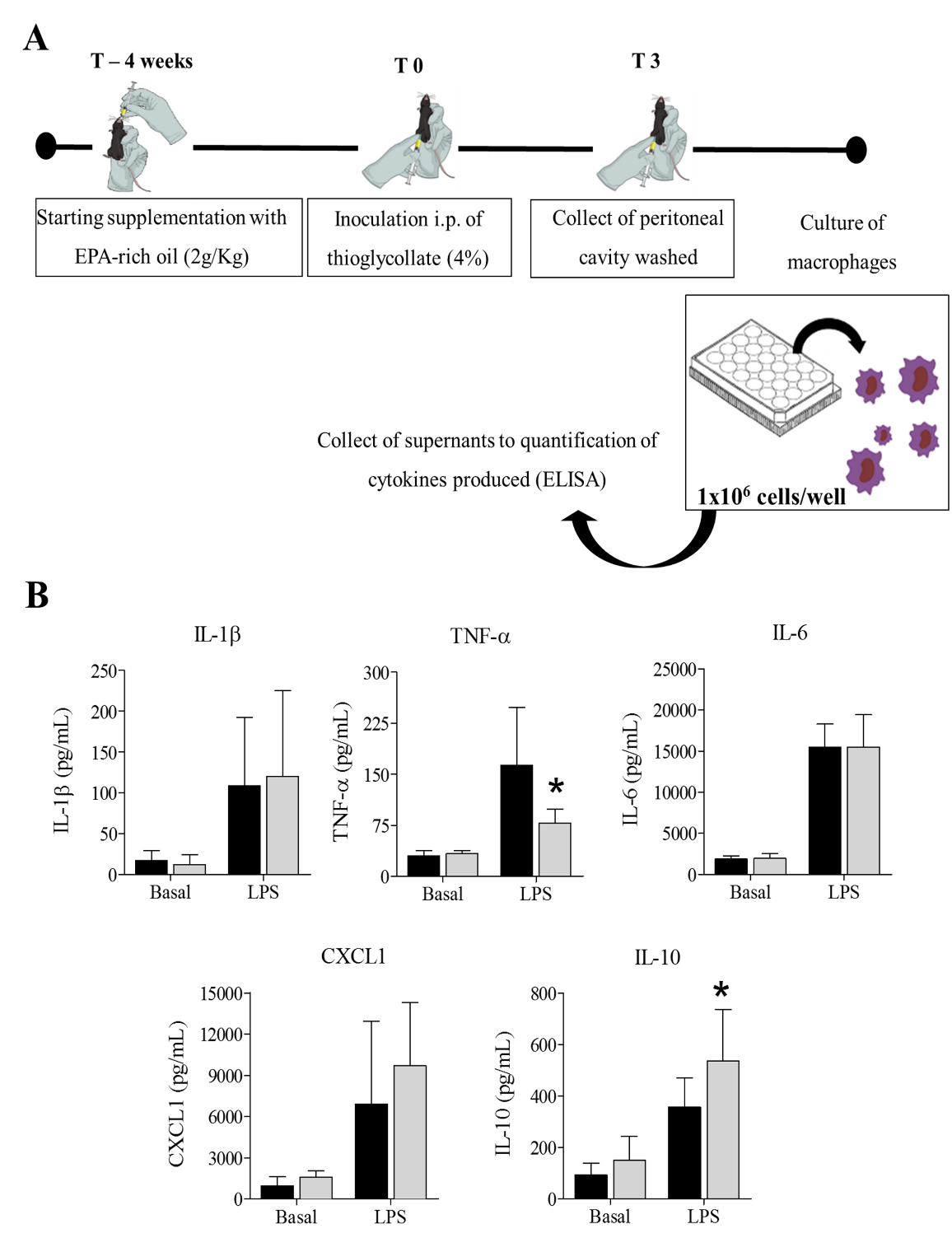


**Supplemental Figure 3. Experimental design and concentrations of cytokines and chemokines in supernatant of macrophages isolated from control mice (C, black line) or EPA treated mice (EPA, grey line) 3 days after inoculation with thioglycolate, and stimulated with LPS (1 μg/mL) in culture for 24 hours.** (A) Experimental design. (B) Interleukin 1-β (IL-1β), interleukin-6 (IL-6), tumor necrosis factor-a (TNF-a), chemokine (C-X-C motif) ligand-1 (CXCL-1) and interleukin-10 (IL-10) were measured. Values were express as mean ± SD (n=4-6 animals/group). (*) p< 0,05 indicates significant differences in relation to control as indicated by Two Way analysis of variance (ANOVA) and Bonferroni posttests.


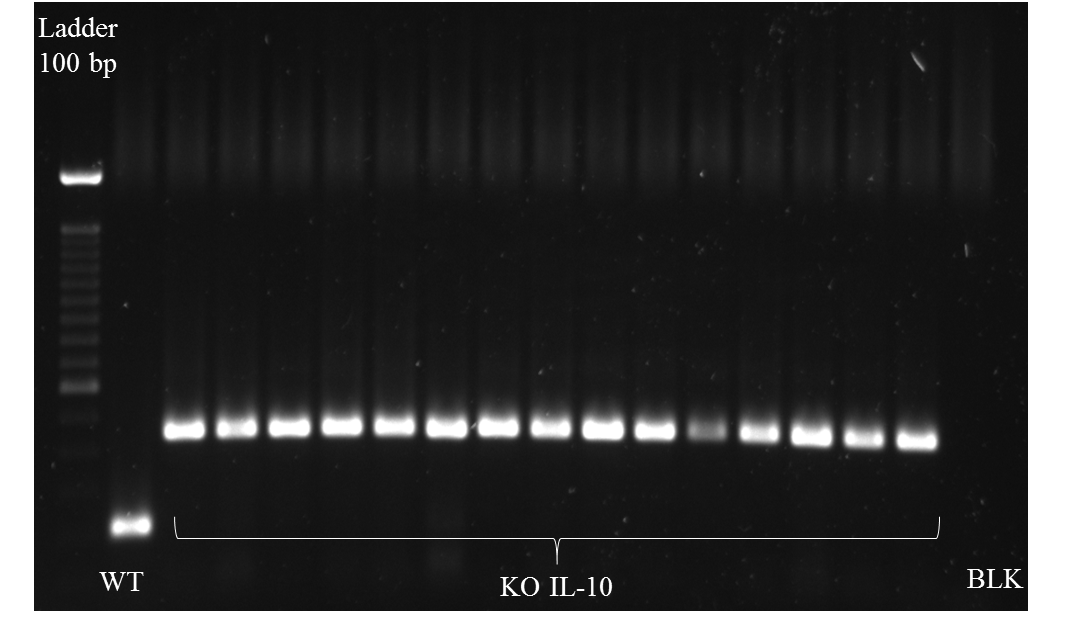


**Supplemental Figure 4.** PCR gel for characterization of IL-10^-/-^ and Control (C57BL / 6) genotypes. Mice that presented a band around 200 bp correspond to the wild pattern (C57BL/6), while the band around 400 bp corresponds to the IL-10^-/-^ (KO) genotype.

**Supplemental Information**

**MATERIALS AND METHODS**

**1.1. Determination of fatty acid composition**

Below, we describe in more details the FAME analysis.

Set up: 30 m x 0.25 μm x 0.25 mm BPX-70 fused silica capillary column with temperature protocol:

o Initial temperature 115°C, hold 2 min, ramp 10°C/min to 200°C, hold 18.5 min, ramp 60°C/min to 245°C, hold 4 min.

o Column: Helium gas, flow rate 1.0, pressure 14.6 and velocity 29.

o Injector: Temperature = 300°C.

o Detector: Hydrogen flow 40.0, air flow 184.0, make up gas Helium, flow 45.0, temperature = 300°C.

Set split ratio to whatever the split ratio was we used for our samples (2:1 for serum and skin samples)

For serum and skin data presented as concentration, a specific internal standard (phosphatidylcholine-PC 15:0 for serum and skin PC extraction and, phosphatidylethanolamine-PE 17:0 for skin PE extraction) was used.

Identification of fatty acid peaks was made by comparison with the 37 FAMEs calibration standard and the area under the peak was used calculate the contribution of individual fatty acids as a percentage of total fatty acids.
